# Supplementary material for: PGC7 Regulates Genome-Wide DNA Methylation by Regulating ERK-Mediated Subcellular Localization of DNMT1
Source: Int J Mol Sci. 2023 Feb 4;24(4):3093. doi: 10.3390/ijms24043093 (PMC9958980; doi:10.3390/ijms24043093)
Supplement: Supplementary file 1 [file ijms-24-03093-s001.zip › ijms-1989369-supplementary.pdf]

International Journal of *Molecular Sciences*

Supplementary Figures

Supplementary Figure S1

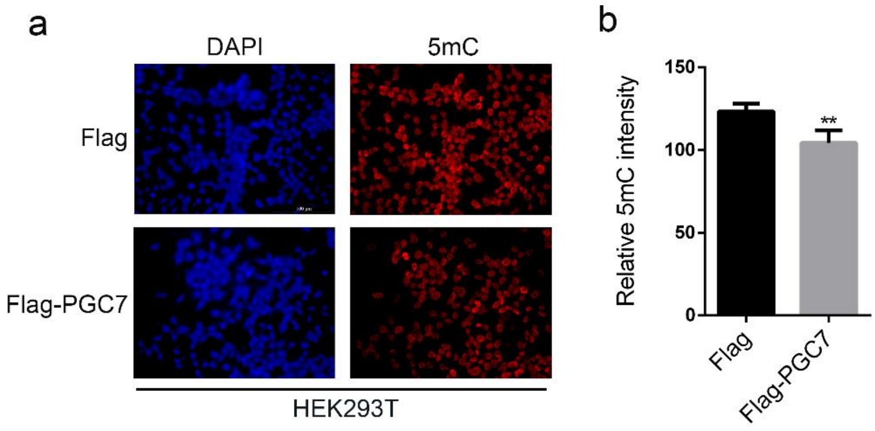

Figure S1. PGC7 regulates genome-wide DNA methylation in HEK293T cells. (a, b) Overexpression of PGC7 reduced genome-wide 5mC levels in HEK293T cells. HEK293T cells were transfected with p3×Flag-CMV-10 or p3×Flag-CMV-10-PGC7 for 36-48 h, after which methylation staining was performed. Image J software was used to determine 5 mC fluorescence intensity, and three or more images were analyzed for both the EV and Flag-PGC7 groups. ** $p < 0.01$.

Supplementary Figure S2

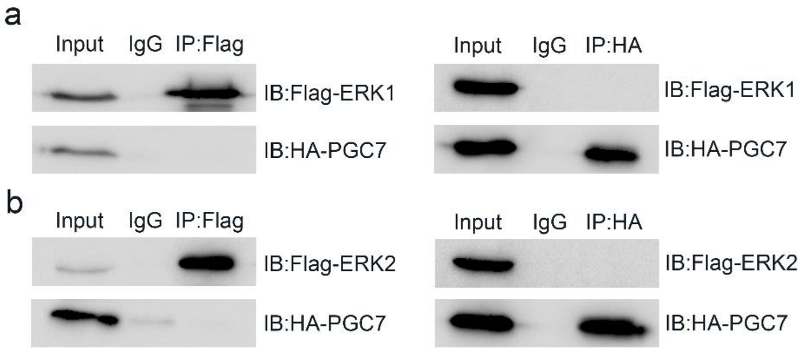

Figure S2. There was no interaction between PGC7 and ERK1 or ERK2 in HEK293T cells. (a, b) HEK293T cells were co-transfected with p3×Flag-CMV-10-ERK1 and pCMV-HA-PGC7 or p3×Flag-CMV-10-ERK2 and pCMV-HA-PGC7. Flag-ERK1 or Flag-ERK2 fusion protein was captured by anti-Flag antibody, and HA-PGC7 fusion protein was captured by anti-HA antibody. Mouse IgG was used as a negative control. A Western blot was used to visualize the expression of ERK1, ERK2, and PGC7 by measuring fluorescent signals linked to anti-Flag and anti-HA antibodies, respectively.

Supplementary Figure S3

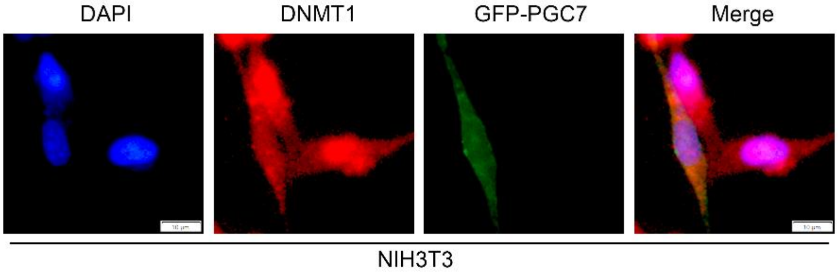

Figure S3. Subcellular co-localization of DNMT1 and PGC7 in NIH3T3 cells. NIH3T3 cells were transfected with pEGFP-C1-PGC7 for immunofluorescence staining.

Supplementary Table S1

Table S1. Quantitative analysis of immunofluorescence in Figure 1b.

	**1**	**2**	**3**	**4**	**5**	**6**	**average**	**P value**
SiNC	89.397	96.957	98.793	100.068	94.437	93.405	95.5095	0.0024
Si*Pgc7*-1	116.92	108.851	100.545	100.497	109.216	109.962	107.6652	

Scheme 2.

Table S2. Quantitative analysis of immunofluorescence in Figure 2b.

	**1**	**2**	**3**	**4**	**average**	**P value**
DMSO	104.015	106.581	104.317	104.971	104.971	0.0191
PD	168.226	170.311	126.083	146.405	152.7563	

Scheme 3. Table S3. Quantitative analysis of protein expression in Figure 2d.

	F9				NIH3T3			
	**1**	**2**	**3**	**average**	**1**	2	3	average
DMSO-ERK	758491	733741	786983	759738.3	610480	617779	640114	622791
PD-ERK	686324	667425	715615	689788	787952	800762	833098	807270.7
DMSO-p-ERK	342168	339240	352245	344551	590902	612216	595001	599373
PD-p-ERK	121412	120026	125508	122315.3	199650	204199	201135	201661.3
DMSO-p-ERK average/DMSO-ERK average	0.453512723				0.962398			
PD-p-ERK average/PD-ERK average	0.177323081				0.249806			
Relative p-ERK level (DMSO/PD)	1/0.390999132				1/0.259566484			

Supplementary Table S4

Table S4. Quantitative analysis of protein expression in Figure 2e.

	F9				NIH3T3			
	**1**	**2**	**3**	**average**	**1**	2	3	average
DMSO-ERK	842363	840435	822899	835232.3	820352	793428	806520	806766.7
1μM PD-ERK	986308	995541	984680	988843	974625	942692	963215	960177.3
2μM PD-ERK	937247	954134	950474	947285	899776	885761	896090	893875.7
DMSO-p-ERK	411570	409235	416802	412535.7	529609	545145	548221	540991.7
1μM PD-p-ERK	210968	211410	213196	211858	435812	446315	450877	444334.7
2μM PD-p-ERK	224852	229429	231843	228708	338412	350811	354398	347873.7
DMSO-p-ERK average/ DMSO-ERK average	0.493917				0.670568			
1μM PD-p-ERK average/ 1μM PD-ERK average	0.214248				0.462763			
2μM PD-p-ERK average/ 2μM PD-ERK average	0.241435				0.389175			
Relative p-ERK level (DMSO/1μM PD/2μM PD)	1/0.433773803/0.488817214				1/0.690106502/0.580365798			
DMSO-GAPDH	1403949	1422970	1366592	1397837	1748641	1763186	1742701	1751509
1μM PD-GAPDH	1622366	1662522	1594499	1626462	1730549	1796172	1715708	1747476
2μM PD-GAPDH	1648381	1686774	1613762	1649639	1391530	1447296	1385895	1408240
DMSO-DNMT1	1139211	1135592	1129056	1134620	2214270	2580383	2548341	2447665
1μM PD-DNMT1	1067168	1068607	1062542	1066106	1630889	1866346	1832361	1776532
2μM PD-DNMT1	746494	749210	746358	747354	1636098	1913252	1874706	1808019
DMSO-DNMT1 average/ DMSO-GAPDH average	0.811697				1.39746			
1μM PD-DNMT1 average/ 1μM PD-GAPDH average	0.655475				1.016627			
2μM PD-DNMT1 average/ 2μM PD-GAPDH average	0.453041				1.283885			
Relative DNMT1 level (DMSO/1μM PD/2μM PD)	1/0.807537/0.558140674				1/0.727482029/0.918727399			

Supplementary Table S5

Table S5. Quantitative analysis of protein expression in Figure 4h.

	Flag-DNMT1WT				Flag-DNMT1S717A			
	1	2	3	average	1	2	3	average
DMSO-IB: Flag	771141	778322	776008	775157	901856	906961	905156	904657.7
PD-IB: Flag	779396	784982	784364	782914	871805	876152	878778	875578.3
DMSO-IB: p-Ser	506707	511043	521674	513141.3	313848	314507	321641	316665.3
PD-IB: p-Ser	434358	440796	454306	443153.3	333048	335706	343161	337305
DMSO-p-Ser average/ DMSO-Flag average	0.661984				0.350039			
PD-p-Ser average/ PD-Flag average	0.566031				0.385237			
Relative p-Ser level (DMSO/ PD)	1/0.855052215/0.528772583/0.581942999							

Supplementary Table S6

Table S6. Quantitative analysis of protein expression in Figure 4i.

	Flag-DNMT1△WT				Flag-DNMT1△S717A			
	1	2	3	average	1	2	3	average
DMSO-IB: Flag	2866420	3033702	2958289	2952804	2651417	2851276	2764297	2755663
PD-IB: Flag	3006836	3242425	3143820	3131027	2608206	2807862	2711903	2709324
DMSO-IB: p-Ser	2725481	2961101	2872321	2852968	2168822	2379002	2298539	2282121
PD-IB: p-Ser	2566965	2814854	2716806	2699542	1890368	2061027	1985927	1979107
DMSO-p-Ser average/ DMSO-Flag average	0.966189				0.828157			
PD-p-Ser average/ PD-Flag average	0.86219				0.73048			
Relative p-Ser level (DMSO/ PD)	1/0.892361745/0.857136978/0.756042447							

Supplementary Table S7

Table S7. Quantitative analysis of immunofluorescence in Figure 5c.

	1	2	3	4	average	P value
SiNC	86.464	94.106	88.727	87.586	89.22075	0.0003
Si*Pgc7*-1	57.444	68.102	69.292	66.486	65.331	

Supplementary Table S8

Table S8. Quantitative analysis of immunofluorescence in Figure S1b.

	1	2	3	4	average	P value
EV	120.292	118.27	126.534	128.23	123.3315	0.0054
Flag-PGC7	103.087	112.067	94.542	107.799	104.3738	

Supplementary Table S9

Table S9. The PCR primers of target gene sequence

Vectors	Primer names	Primer sequences (5′-3′)
p3×Flag-CMV-10	ERK1-Forward	CCCAAGCTTGGAGGAGTGGAGATGGCG
	ERK1-Reverse	CGGGATCCTTAGGGGCCCTCTGGCGC
	ERK2-Forward	CCCAAGCTTTGTGCAGCCAACATGGCG
	ERK2-Reverse	CGGGATCCTTAAGATCTGTATCCTGGCTGG
	MEK1-Forward	CCCAAGCTTATGCCCAAGAAGAAGCCG
	MEK-Reverse	CGGGATCCTCAGATGCTGGCAGCGTG
	DNMT1-Forward	CCCAAGCTTCTGTCGTCTGCAACCTGCAA
	DNMT1-Reverse	GGGGTACCGGGTGAGAGCACTAGTCCTTGG
	DNMT1S717A-Forward	GTGTCAGAGATGCCAGCACCCAAAAAGCTGCATC
	DNMT1S717A-Reverse	GATGCAGCTTTTTGGGTGCTGGCATCTCTGACA
	DNMT1S958A-Forward	CAACATCAAAGTGGCTGCCCCCGTGAAACGCCC
	DNMT1S958A-Reverse	GGGCGTTTCACGGGGGCAGCCACTTTGATGTTG
	DNMT1S1421A-Forward	CTGCAAGGACATGGCCCCACTGGTGGCTGCCCG
	DNMT1S1421A-Reverse	CGGGCAGCCACCAGTGGGGCCATGTCCTTGCAG
	PGC-Forward	CCCAAGCTTATGGAGGAACCATCAGAG AAAGTCGACCCAATGAAGGACCCT
	PGC7-Reverse	GGAATTCCTAATTCTTCCCGATTTTCGC
pCMV-HA	ERK1-Forward	CGGAATTCGGGGAGGAGTGGAGATGGCG
	ERK1-Reverse	CCGCTCGAGTTAGGGGCCCTCTGGCGC
	PGC7-Forward	CCCAAGCTT ATGGAGGAACCATCAGAGAAA
	PGC7-Reverse	GGGGTACCCTAATTCTTCCCGATTTTCGCAT
pEGFP-C1	PGC7-Forward	GGAATTCTATGGAGGAACCATCAGAGAAAGTC
	PGC7-Reverse	TCGGTACCCTAATTCTTCCCGATTTTCGC

Supplementary Table S10

Table S10. Specific siRNA oligonucleotides for Pgc7

Name	Sequence (5′-3′)
Si*Pgc7-1*	GCACAACGAUCCAGAUUUA
Si*Pgc7-2*	AAAGGCUCGAAGGAAAUGAGUU
siRNA-NC	UUCUCCGAACGUGUCACGUTT

Supplementary Table S11

Table S11. The qPCR primers of target gene

Primer names	Primer sequences (5′-3′)
Mek1-Forward	TGCTGAGTTGCAGGCTCTT
Mek1-Reverse	ATCCTTCAGTTCCCCCACCT
Mek2-Forward	GTCCGCCTTCCACCTTCT
Mek2-Reverse	GCTCATCCAGGTCCAGCTC
Erk1-Forward	CATCCGAGACATCCTCAGAGC
Erk1-Reverse	CGCAGGTGGTGTTGATAAGC
Erk2-Forward	TGAAGTTGAACAGGCTCTGGC
Erk2-Reverse	TGAGCCCTTGTCCTGACCAAT
Gapdh-Forward	TGTGAGGGAGATGCTCAGTG
Gapdh-Reverse	TGTTCCTACCCCCAATGTGT
Pgc7-Forward	AAAGCGCCTTTCCCAAGAGA
Pgc7-Reverse	TGGCAGAAAGTGCAGAGACA
